# Successful Hydrogel Spacer Placement for Salvage Radiation Therapy After Focal High-Intensity Focused Ultrasound for Prostate Cancer: A Case Report

**DOI:** 10.7759/cureus.92573

**Published:** 2025-09-17

**Authors:** Shuhei Ishii, Koyo Kikuchi, Takehiro Aoyagi, Yukihisa Owari, Wataru Obara

**Affiliations:** 1 Department of Urology, Iwate Medical University, Shiwa-gun, JPN; 2 Department of Radiation Oncology, Iwate Medical University, Shiwa-gun, JPN; 3 Department of Urology, Iwate Prefectural Miyako Hospital, Miyako, JPN; 4 Department of Urology, Iwate Medical University Hospital, Shiwa-gun, JPN

**Keywords:** androgen deprivation therapy, high-intensity focused ultrasound ablation, hydrogels, organs at risk, prostatic neoplasms, radiotherapy, salvage therapy

## Abstract

After focal high-intensity focused ultrasound (HIFU) for prostate cancer, evidence on the safety of hydrogel spacer placement remains limited. A 65-year-old man who had undergone focal HIFU for low-risk localized prostate cancer two years earlier presented with local recurrence (prostate-specific antigen (PSA) 6.5 ng/mL, rT1cN0M0, Gleason scores 4+3 and 3+4) and was referred for salvage radiation therapy. Hydrogel spacer placement was planned before radiation therapy. The patient was placed in the lithotomy position under sacral block and local anesthesia, and the procedure was performed under transrectal ultrasound guidance. Using a transperineal approach, saline was injected into the dorsal aspect of Denonvilliers’ fascia for hydrodissection, during which moderate tissue resistance was noted. However, no significant adhesions were identified, and adequate separation between the prostate and rectum was achieved. The hydrogel spacer was then successfully inserted without procedure-related complications. External beam radiation therapy (60 Gy in 20 fractions) was completed without early adverse events. This case demonstrates that salvage radiation therapy with hydrogel spacer placement can be safely performed in patients with prostate cancer previously treated with focal HIFU.

## Introduction

Focal therapies such as high-intensity focused ultrasound (HIFU) for localized prostate cancer aim to minimize adverse events while maintaining oncologic outcomes [1]. Although toxicity is generally lower, local recurrence may necessitate salvage radiation therapy [1]. Hydrogel spacers are biocompatible materials injected into the perirectal space to physically separate the prostate from the rectum, thereby reducing the rectal radiation dose. They are widely used to minimize rectal toxicity during prostate radiation therapy, and their safety when placed before primary treatment has been well established [2]. In contrast, evidence on the safety of hydrogel spacer placement before salvage radiation therapy following HIFU is limited. We report a case in which a hydrogel spacer was safely placed before salvage radiation therapy after focal HIFU and discuss the procedural considerations and clinical implications of this approach.

## Case presentation

A 65-year-old man was referred to our hospital for salvage radiation therapy after local recurrence following focal HIFU for prostate cancer performed at another institution.

The prior clinical course was as follows: in December 2019, a prostate biopsy was performed for a prostate-specific antigen (PSA) level of 6.634 ng/mL, and the patient was diagnosed with low-risk, organ-confined prostate adenocarcinoma (cT2aN0M0, Gleason score 3+3). In March 2020, he underwent focal HIFU under general anesthesia. Treatment was performed using the Sonablate 500 system (SonaCare Medical, Charlotte, NC, USA) with real-time transrectal ultrasound (TRUS) guidance, and power settings were adjusted between 22 and 48 W. The focal zone dimensions were 3 × 3 × 12 mm for a focal length of 40 mm and 3 × 3 × 10 mm for a focal length of 30 mm. The targeted areas included the right transition zone (TZ) and the lateral portion of the left peripheral zone (PZ). The PSA level declined to a nadir of 3.80 ng/mL in December 2020 after focal HIFU. Subsequently, it showed a gradual increase, reaching 5.97 ng/mL by December 2021. In January 2022, a PSA level of 6.5 ng/mL prompted repeat biopsy, which demonstrated adenocarcinoma (Gleason scores 4+3=7 and 3+4=7) in the left TZ and PZ. Based on these findings, local recurrence in the prostate was diagnosed (rT1cN0M0). Androgen deprivation therapy (ADT) with leuprorelin acetate and bicalutamide was initiated; however, bicalutamide was later discontinued because of hepatotoxicity.

**Figure 1:**
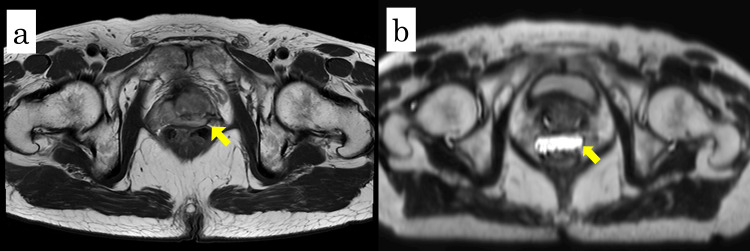
Pre- and post-spacer MRI findings (a) T2-weighted axial magnetic resonance imaging (MRI) before spacer placement showing close approximation between the prostate and rectum (yellow arrow). (b) T2-weighted axial MRI after spacer placement demonstrating a hyperintense area (yellow arrow) corresponding to the hydrogel spacer, indicating adequate separation.

In September 2024, the patient was referred to our hospital for salvage radiation therapy. At referral, the radiation oncologist proposed fiducial marker and hydrogel spacer placement to facilitate image-guided radiation therapy and optimize rectal dose reduction. Because of the limited reports on hydrogel spacer placement after HIFU, preoperative prostate MRI was performed to assess the feasibility and safety of the procedure. T2-weighted MRI demonstrated close apposition of the prostate and rectum without evidence of adhesion, as shown in Figure 1a. Spacer placement was therefore planned before initiating radiation therapy.

**Figure 2:**
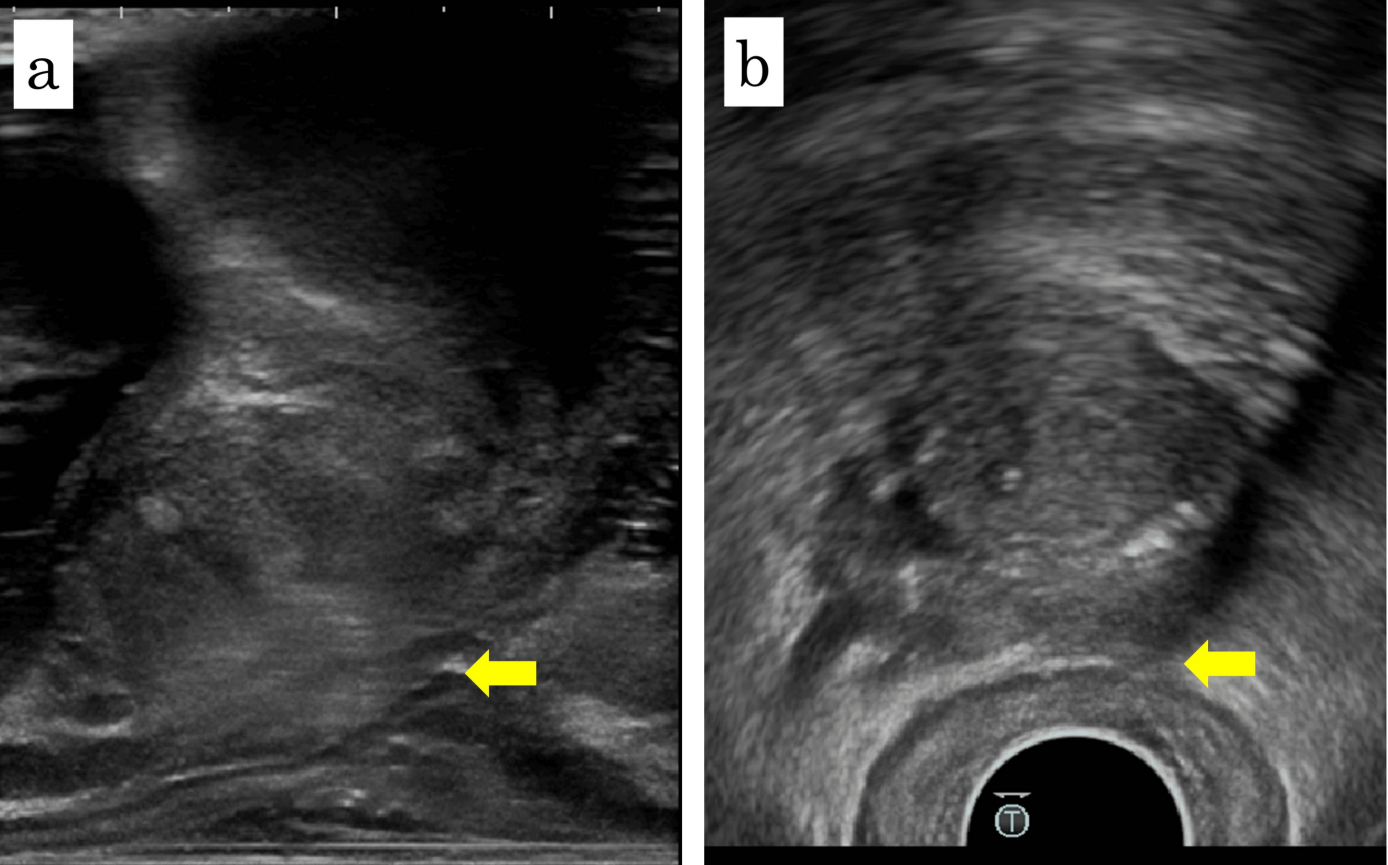
Sagittal and axial TRUS images before spacer placement Sagittal (a) and axial (b) transrectal ultrasound (TRUS) images obtained immediately before spacer placement. Both views show a thin, well-defined Denonvilliers’ fascia (yellow arrows) without evidence of fibrosis or adhesion between the posterior prostate and anterior rectal wall.

**Figure 3:**
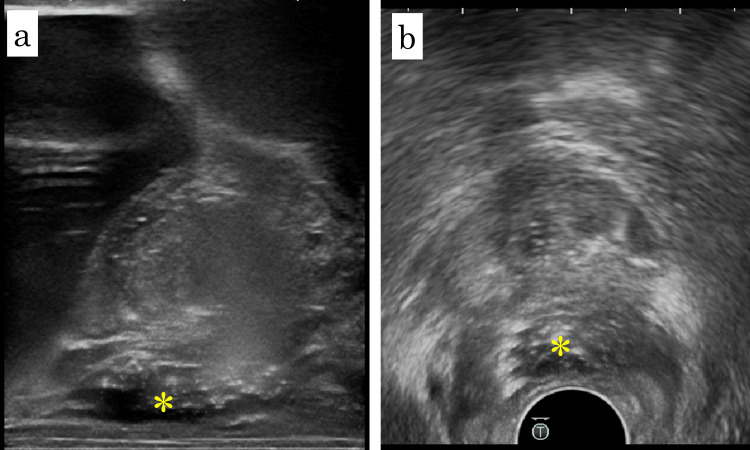
Sagittal and axial TRUS images after spacer placement Sagittal (a) and axial (b) transrectal ultrasound (TRUS) images obtained after hydrogel spacer placement. The hydrogel (yellow asterisk) is visible between the posterior prostate and anterior rectal wall, confirming successful separation.

Hydrogel spacer placement (SpaceOAR; Boston Scientific, Marlborough, MA, USA) was performed in December 2024 with the patient in the lithotomy position under sacral block and local anesthesia. Sagittal and axial TRUS images showed a clearly visualized Denonvilliers’ fascia without significant adhesion, as shown in Figure 2. Two gold fiducial markers were first placed transperineally, one in each lobe of the prostate. A puncture needle was then advanced transperineally toward the dorsal aspect of Denonvilliers’ fascia. Although moderate resistance was encountered, the needle tip was advanced to the prostate base. Hydrodissection with normal saline was performed. Although moderate resistance was noted in part of the right basal prostate, adequate separation was ultimately achieved. As shown in Figure 3, 10 mL of hydrogel spacer was injected into the created space. The procedure was completed within 10 minutes without complications. Post-procedural T2-weighted MRI confirmed appropriate spacer placement, as shown in Figure 1b. External beam radiation therapy (60 Gy in 20 fractions with volumetric modulated arc therapy) was subsequently completed without early adverse events. At seven months post-treatment, the PSA level remained undetectable.

## Discussion

Focal therapy is an emerging treatment option for localized prostate cancer; however, its optimal indication remains debated. According to the American Urological Association [3] and National Comprehensive Cancer Network [4] guidelines, focal therapy with HIFU is not routinely recommended for patients in any risk category because of insufficient supporting evidence. Nonetheless, some patients prefer focal therapy over active surveillance, prioritizing quality-of-life preservation compared with radical treatment [5]. The seven-year failure-free survival rate after focal HIFU has been reported as 69%, and the need for salvage therapy remains an important concern [2]. In cases of biochemical recurrence, standard curative options include external beam radiation therapy or radical prostatectomy [6]. In the present case, the patient - initially treated with focal HIFU for low-risk prostate cancer - developed local recurrence and subsequently underwent external beam radiation therapy combined with ADT.

Rectal bleeding is a recognized complication of radiation therapy for prostate cancer. To mitigate this risk, a hydrogel spacer was developed to physically separate the prostate and rectum. Severe complications from spacer placement are rare, and its use has been shown to reduce rectal radiation dose and significantly decrease the risk of late rectal toxicity [7-9]. Specifically, Hamstra et al. demonstrated that patients in the spacer group had significantly lower rectal toxicity at three years (grade ≥1, 9.2% vs 2.0%; grade ≥2, 5.7% vs 0%) and better bowel quality of life (QOL) outcomes compared with the control group [8]. Some studies have suggested that salvage radiotherapy after HIFU may be associated with increased urinary toxicity compared with primary radiotherapy [10,11], whereas others have reported that it can be performed safely [12,13], with no clear evidence of increased rectal toxicity. In this case, we elected to place a hydrogel spacer to further minimize the risk of radiation-induced rectal complications. There are a few reports describing the use of hydrogel spacers in salvage radiation therapy for recurrent prostate cancer [14,15]. In these reports, hydrogel spacer placement was performed safely and proved useful in minimizing rectal toxicity from brachytherapy.

Although the patient had a history of HIFU, hydrogel spacer placement was performed after pre-procedural MRI assessment and detailed evaluation of Denonvilliers’ fascia under ultrasound guidance. Moderate resistance was encountered during hydrodissection; however, adequate separation between the prostate and rectum was achieved, enabling safe spacer placement.

This report has certain limitations. First, as a single case report, it is difficult to generalize the efficacy and safety of hydrogel spacer placement after HIFU. Although the procedure was successful in this case, further case accumulation and evaluation of outcomes in larger cohorts are warranted. Second, the technical difficulty of spacer placement may depend on the site of focal ablation with HIFU. When the thermal coagulation effect is concentrated in the posterior prostate, severe fibrosis and adhesion of Denonvilliers’ fascia may develop, making placement challenging. After HIFU targeting the prostate base or posterior region, achieving uniform spacer distribution may also be difficult. Third, if spacer placement is technically challenging, the procedure should be discontinued rather than forcibly continued. If optimal deployment cannot be achieved or if severe posterior prostatic fibrosis prevents adequate hydrodissection, it will lead to inappropriate hydrogel spacer placement. Criteria for patient selection and procedural guidelines for hydrogel spacer placement after HIFU have not yet been established, underscoring the need for further research.

## Conclusions

This case demonstrates that salvage radiation therapy with hydrogel spacer placement can be safely performed in patients with prostate cancer previously treated with focal HIFU. Future accumulation of similar cases and evaluation of outcomes are expected to provide more robust evidence regarding the success rate of spacer placement and the clinical value of salvage radiotherapy combined with hydrogel spacer use after HIFU.
